# Supplementary material for: Upgrading the Properties of Reduced Graphene Oxide and Nitrogen-Doped Reduced Graphene Oxide Produced by Thermal Reduction toward Efficient ORR Electrocatalysts
Source: Nanomaterials (Basel). 2019 Dec 11;9(12):1761. doi: 10.3390/nano9121761 (PMC6956339; doi:10.3390/nano9121761)
Supplement: Supplementary file 1 [file nanomaterials-09-01761-s001.pdf]

nanomaterials

Article

Upgrading the Properties of Reduced Graphene Oxide and Nitrogen-Doped Reduced Graphene Oxide Produced by Thermal Reduction toward Efficient ORR Electrocatalysts

Carolina S. Ramirez-Barria [1,2], Diana M. Fernandes [3,*], Cristina Freire [3], Elvira Villaro-Abalos [4], Antonio Guerrero-Ruiz [2,*] and Inmaculada Rodríguez-Ramos [1,*]

1 Instituto de Catálisis y Petroleoquímica, CSIC, Marie Curie 2, 28049 Madrid, Spain; ramirez.carolina@outlook.com
2 Dpto. Química Inorgánica y Técnica, Facultad de Ciencias UNED, Senda del Rey 9, 28040 Madrid, Spain
3 REQUIMTE/LAQV, Departamento de Química e Bioquímica, Faculdade de Ciências, Universidade do Porto, 4169-007 Porto, Portugal; acfreire@fc.up.pt
4 Interquimica, Antonio de Nebrija 8, 26006 Logroño, Spain; evillaro@interquimica.org
* Correspondence: diana.fernandes@fc.up.pt (D.M.F.); aguerrero@ccia.uned.es (A.G.-R.); irodriguez@icp.csic.es (I.R.-R.); Tel.: +34 915854765 (I.R.-R.)

1. Experimental

1.1. Materials characterization methods

The chemical analysis (C, H and N) of the prepared materials were determined using a Perkin-Elmer elemental analyser. The textural characterization and surface area (S_{BET}) determinations were obtained from the nitrogen adsorption (-196 ºC) isotherms, which were obtained using an ASAP model 2020 instrument. To obtain the point of zero charge (PZC) of graphenic materials, the electrophoretic mobility (μ) vs. pH of the samples was measured in a Zeta Meter 3.0+ at 298 K. PZC was determined following the experimental procedure described in detail elsewhere.[i] The morphology of the samples was observed using a scanning electron microscope (SEM, HitachiTM-100) and a field emission transmission electron microscopy (TEM, JEOL JEM-2100F microscope at 200 kV). For the TEM study the samples were ultrasonically suspended in ethanol before deposition over a copper grid coated with holey carbon layers (Aname, Lacey carbon 200 mesh). Average particle size was calculated based on a minimum of 200 particles.

The samples were also analysed by X-ray diffraction (XRD), using a Polycristal X'Pert Pro PANalytical diffractometer with Ni-filtered Cu/K radiation (λ = 1.54 Å) operating at 45 kV and 40 mA. For each sample, Bragg's angles between 4◦ and 90◦ were scanned at a rate of 0.04°/s. Thermal Analysis were also recorded in a CI Electronics microbalance MK2–MC5 under air. All the samples were analysed by X-ray photoelectron spectroscopy (XPS) using aan SPECS GmbH with UHV system, energy analyser PHOIBOS 150 9MCD, which operated with a monochromatized Al K_{α} source (1486.71 eV). The binding energies (BE) were referenced to the C 1s peak at 284.6 eV of the graphitic material used as an internal standard. The estimated equipment error in the energy determinations is less than 0.01 eV. The spectral data for each sample were analysed using CASA XPS software. Microscopic confocal Raman spectrometer (Renishaw inVia, 532 nm laser) was also employed for the characterization of graphene materials. Over each material there were acquired 25 spectra in different samples points. Average and components fitting was obtained using Wire 4.2 software.

1.2. ORR electrochemical performance

Before any type of modification, a cleaning procedure was performed to the RDE with diamond polishing pastes of 6, 3 and 1 μM (Buehler) on a microcloth pad (BAS), followed by washing with ultra-pure water (18.2 MΩ cm at 25°C, Millipore).

The effective ORR current was obtained by subtracting the current obtained in N_2-saturated electrolyte by that obtained in O_2-saturated.

Even though the potential were measured against the Ag/AgCl reference electrode these were converted to the reversible hydrogen electrode (RHE) using the Eq. 1 for a proper comparison with the literature results.

$$E_{(RHE)} = E_{(Ag/AgCl)} + 0.059 \text{ pH} + E^{o}_{(Ag/AgCl)}, \tag{1}$$

where $E_{(RHE)}$ is the potential vs. RHE, $E^{o}_{(Ag/AgCl)}$ = 0.1976 V (25 ºC) and $E_{(Ag/AgCl)}$ is the potential measure vs. Ag/AgCl.[ii]

Onset potential (E_{onset}) was calculated as described in literature and is defined as the potential at which the reduction of O_2 beggins.[2]

The kinetic parameters and the number of electrons transferred per O_2 molecule (n_{O2}) in the oxygen reduction reaction were determined using the following Koutecky-Levich equations:

$$\frac{1}{j} = \frac{1}{j_L} + \frac{1}{j_k} = \frac{1}{B\omega^{1/2}} + \frac{1}{j_k}, \tag{2}$$

$$B = 0.2\, n_{O2}\, F\, (D_{O2})^{2/3}\, v^{-1/6}\, C_{O2}, \tag{3}$$

Here, j is the current density measured, j_L and j_k are the diffusion-limiting and kinetic current densities, ω is the angular velocity, F is the Faraday constant (96 485 C mol^{-1}), D_{O2} is the O_2 diffusion coefficient (1.95×10^{-5} cm^2 s^{-1}), v is the electrolyte kinematic viscosity (0.008977 cm^2 s^{-1}), C_{O2} is the O_2 bulk concentration (1.15×10^{-3} mol dm^{-3}). For rotation speeds in rpm is adopted a constant of 0.2.[iii]

Tafel plots were obtained after the measured LSV currents were corrected for diffusion to yield the corresponding kinetic current values. The j_L parameter, obtained through the combination of Eq. 2 and 3, was used to make the mass transport correction. The values of j_k obtained were normalized for the total deposited mass of EC.

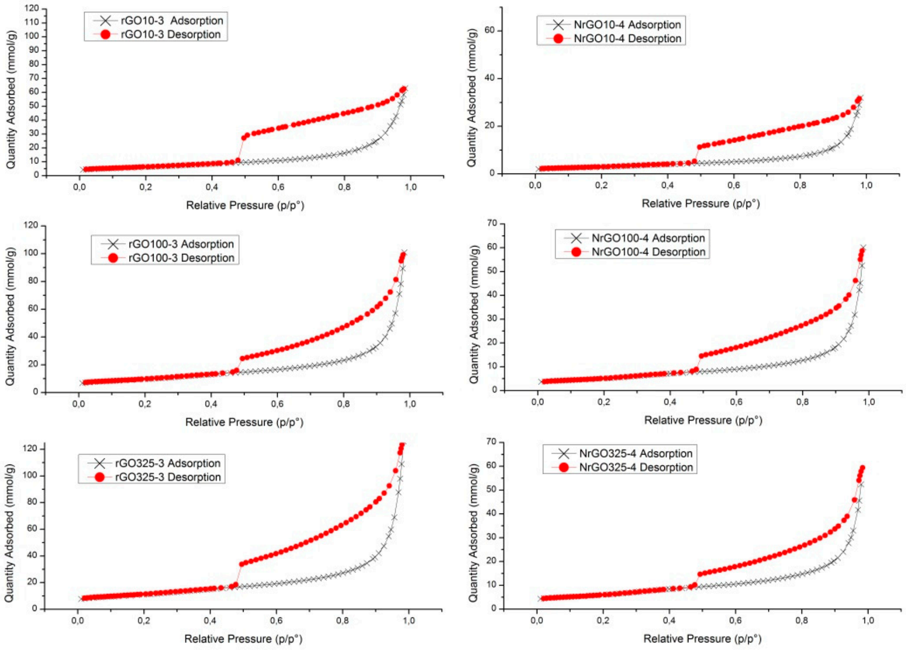

Figure S1. N_2 adsorption–desorption isotherm for samples rGO_{325}-3, $NrGO_{325}$-4, rGO_{100}-3, $NrGO_{100}$-4, rGO_{10}-3 and $NrGO_{10}$-4.

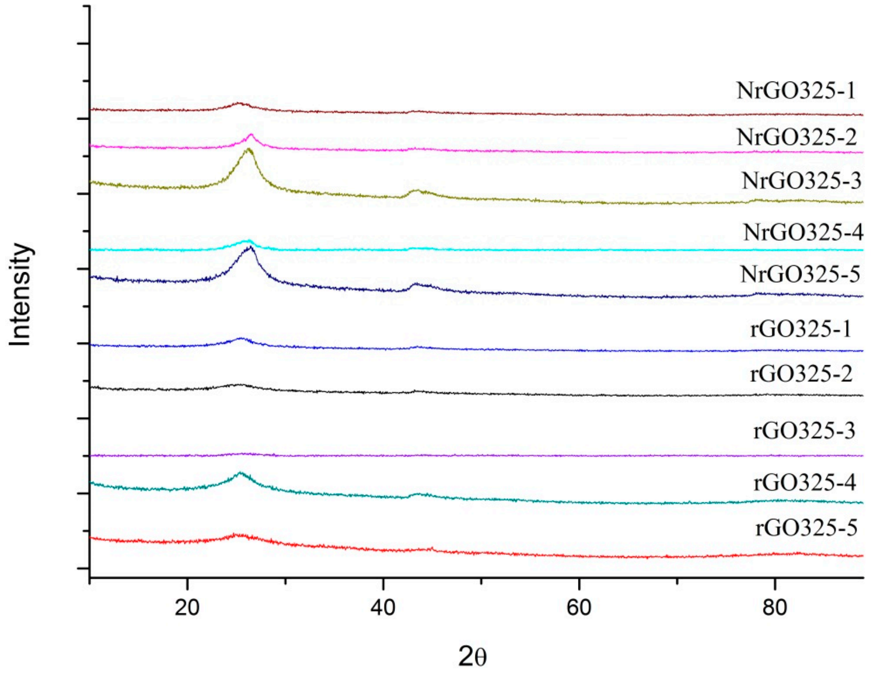

Figure S2. XRD patterns of graphenic materials: $NrGO_{325}$-1 $NrGO_{325}$-2, $NrGO_{325}$-3, $NrGO_{325}$-4, $NrGO_{325}$-5, rGO_{325}-1, rGO_{325}-2, rGO_{325}-3, rGO_{325}-4 and rGO_{325}-5.

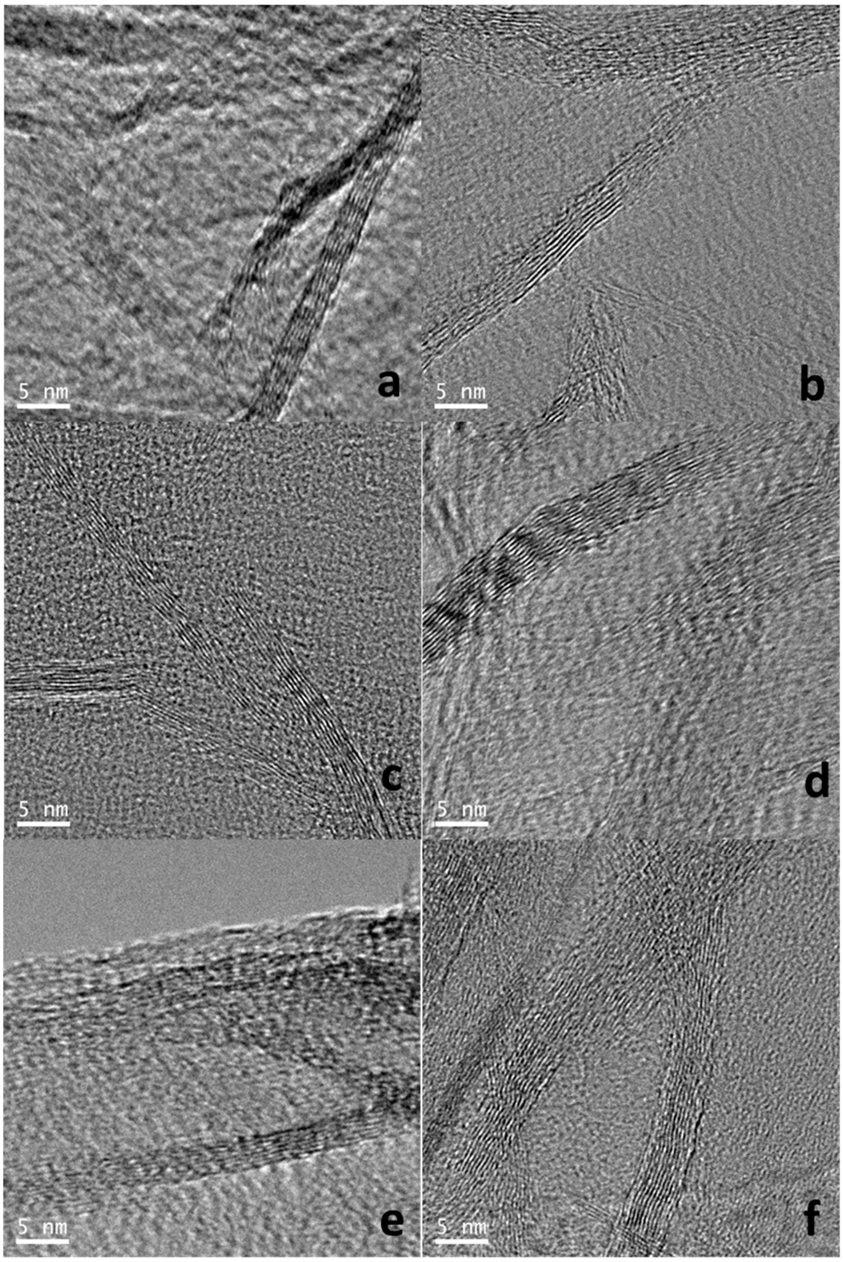

Figure S3. TEM micrograph for (a) rGO_{325}-3, (b) $NrGO_{325}$-4, (c) rGO_{100}-3, (d) $NrGO_{100}$-4, (e) rGO_{10}-3 and (f) $NrGO_{10}$-4.

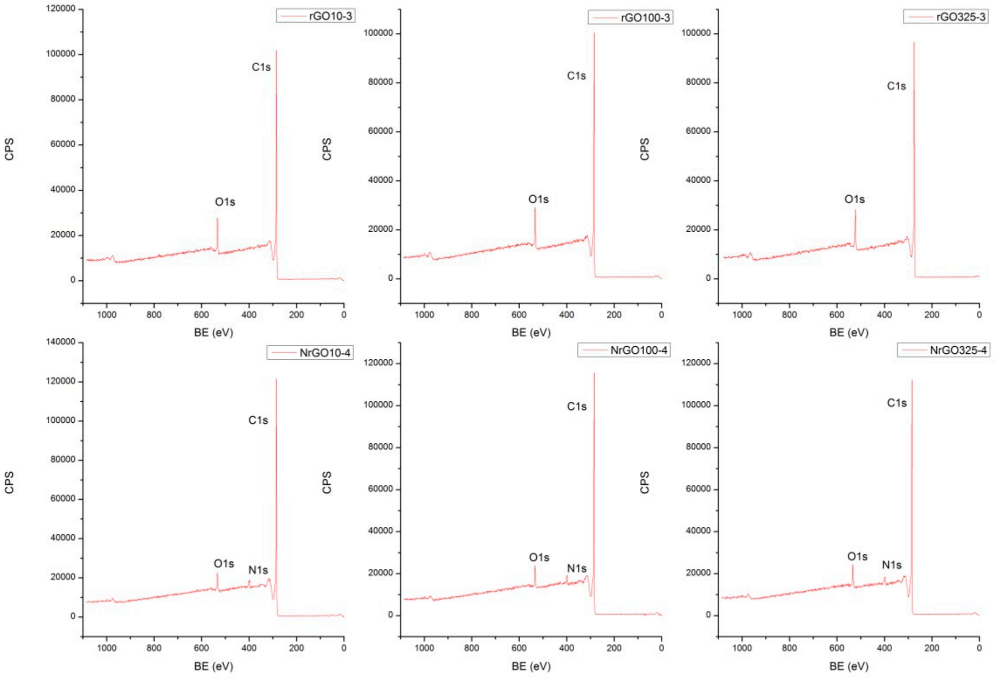

Figure S4. Survey XPS spectra for rGO_{325}-3, $NrGO_{325}$-4, rGO_{100}-3, $NrGO_{100}$-4, rGO_{10}-3 and $NrGO_{10}$-4.

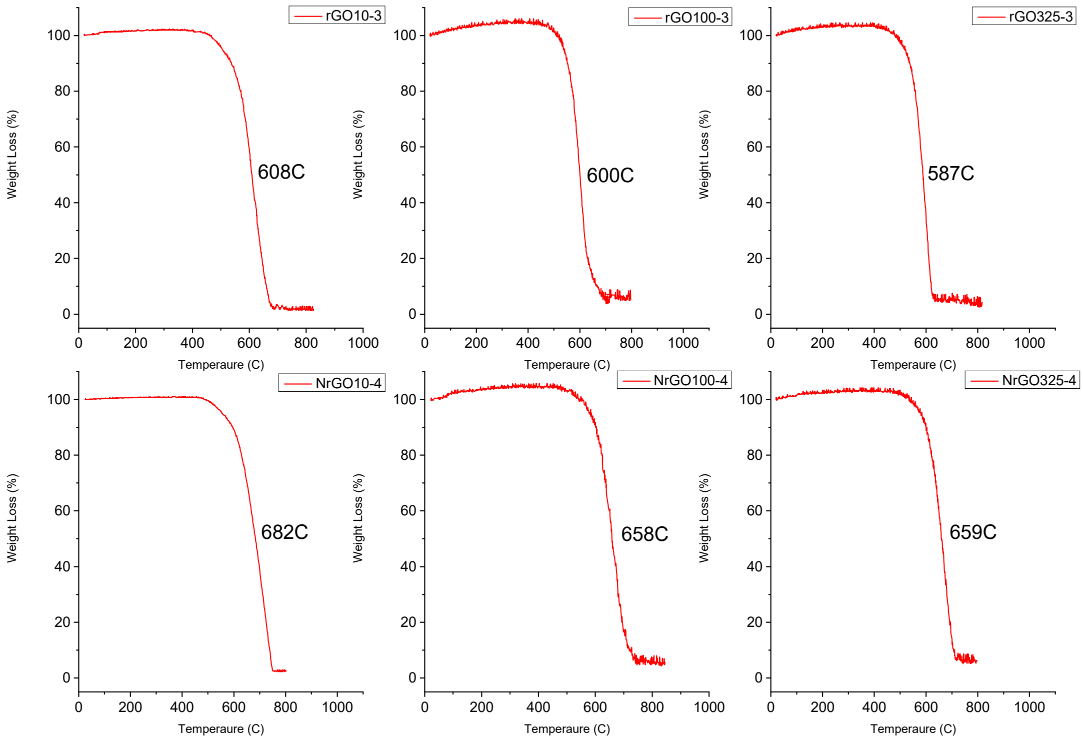

Figure S5. TG analysis under air for rGO_{m}-3 and $NrGO_{m}$-4 samples.

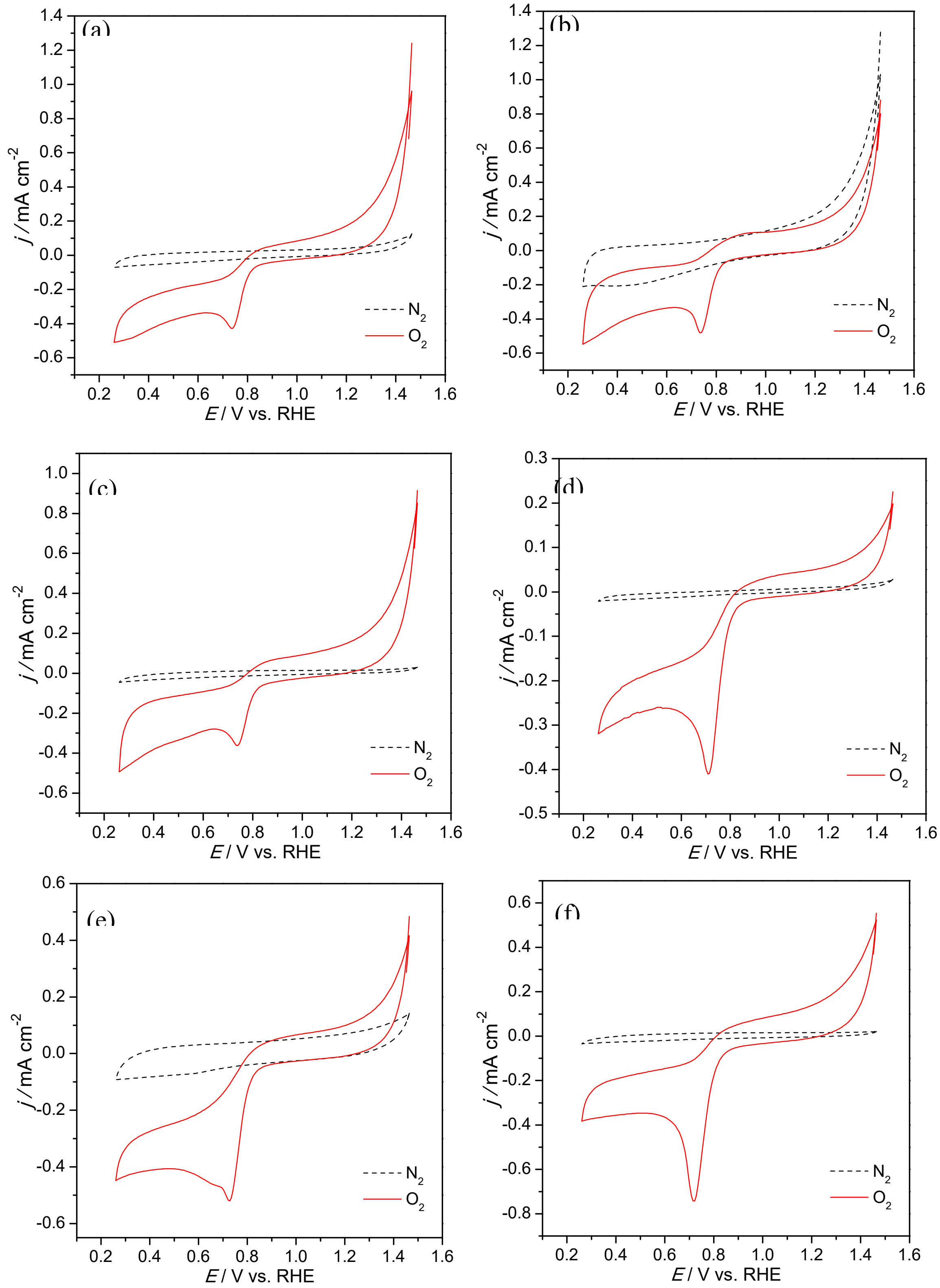

Figure S6. CVs of rGO_{10}-3 (a), rGO_{100}-3 (b), rGO_{325}-3 (c), $NrGO_{10}$-4 (d), $NrGO_{100}$-4 (d) and $NrGO_{325}$-4 (f) modified electrodes in N_2-saturated (dash line) and O_2-saturated (full line) 0.1 mol dm^{-3} KOH solution at 0.005 V s^{-1}.

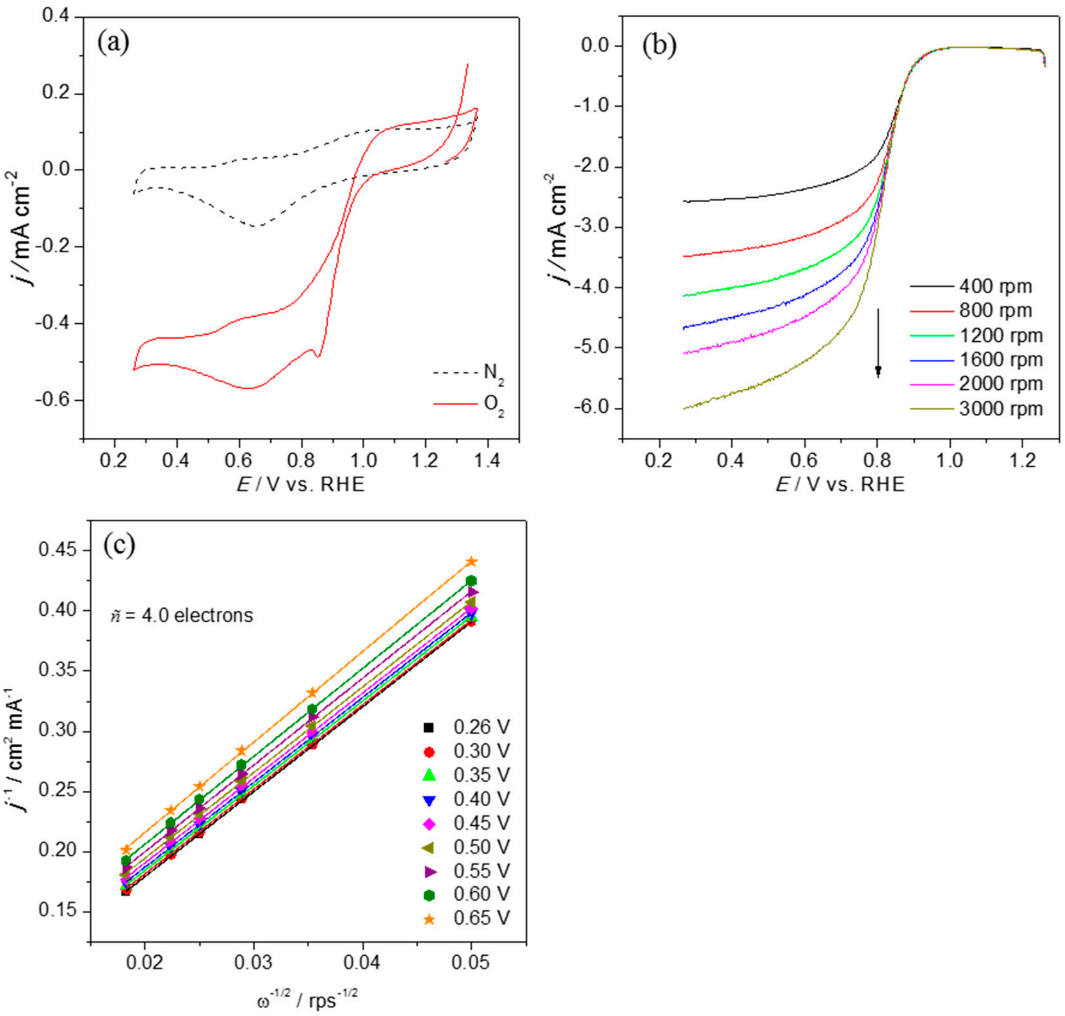

Figure S7. CVs of commercial Pt/C (20 wt %) modified electrode in N_2-saturated (dash line) and O_2-saturated (full line) 0.1 mol dm^{-3} KOH solution at 0.005 V s^{-1} (a), ORR polarization curves at different rotation rates in O_2-saturated 0.1 mol dm^{-3} KOH solution at 0.005 V s^{-1} (b) and the corresponding Koutecky-Levich (K-L) plots (c).

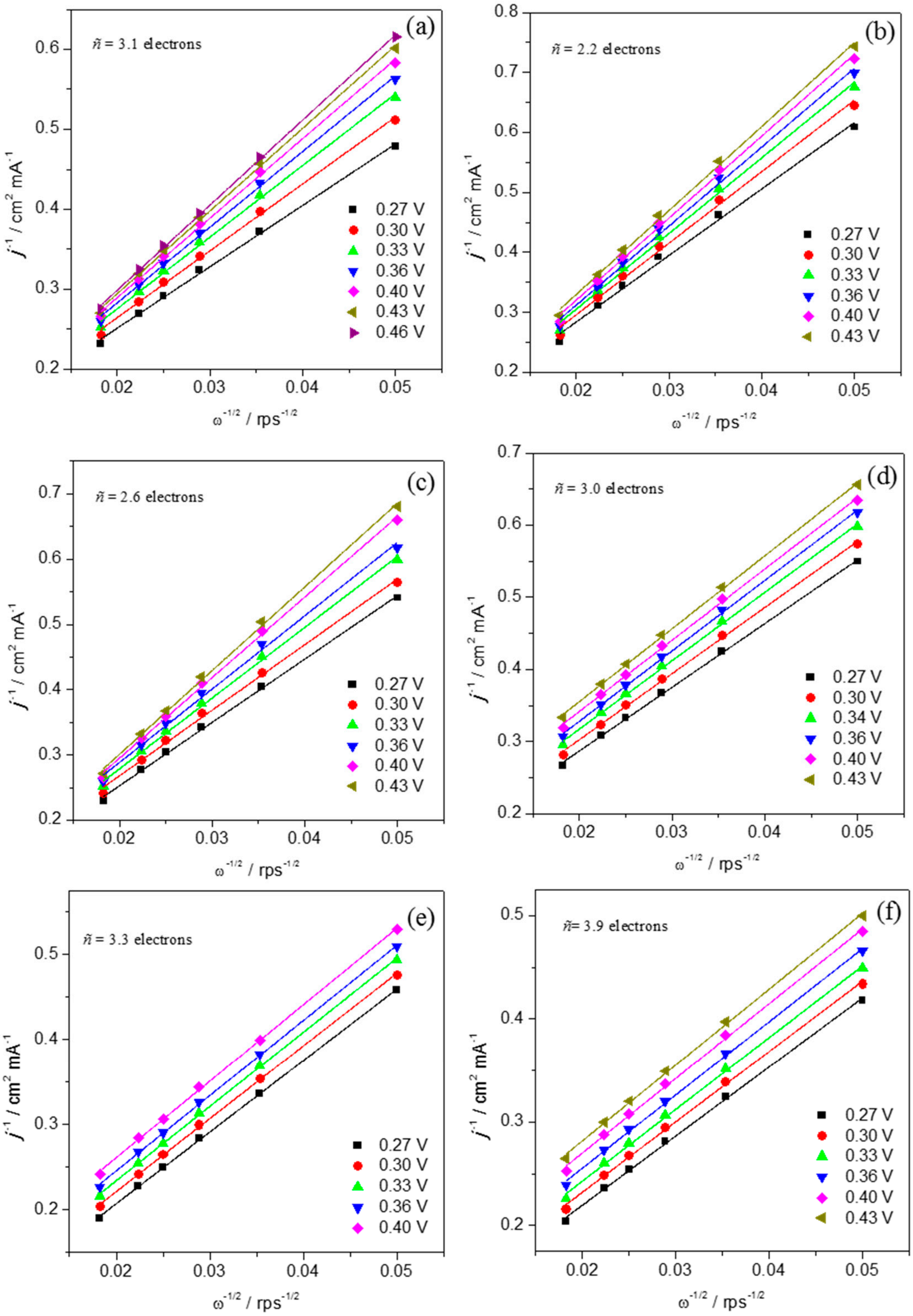

Figure S8. Koutecky-Levich (K-L) plots of rGO_{10}-3 (a), rGO_{100}-3 (b), rGO_{325}-3 (c), $NrGO_{10}$-4 (d), $NrGO_{100}$-4 (e) and $NrGO_{325}$-4 (f) modified electrodes obtained from data in Figure S8.

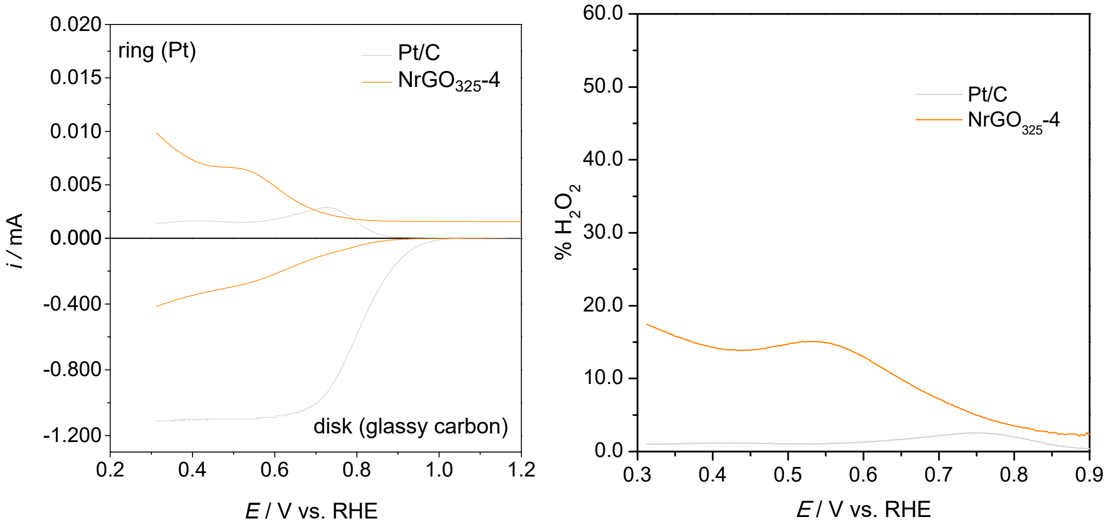

Figure S9. Linear sweep voltammograms recorded with RRDE in O_2-saturated 0.1 mol dm^{-3} KOH solution, at 1600 rpm and v = 0.005 V s^{-1} and the ring potential was kept at E = 1.16 V vs. RHE (a); Estimated percentage of H_2O_2 formed (b).

References

1. C. Ramirez-Barria, A. Guerrero-Ruiz, E. Castillejos-Lopez, I. Rodríguez-Ramos, J. Durand, J. Volkman, P. Serp, Surface properties of amphiphilic carbon nanotubes and study of their applicability as basic catalysts, *RSC Adv*. **2016**, 6, 54293–54298.
2. C. Freire, D.M. Fernandes, M. Nunes, V.K. Abdelkader, POM & MOF-based Electrocatalysts for Energy-related Reactions, *ChemCatChem*, **2018**, 10, 1703–1730.
3. G. Tuci, C. Zafferoni, A. Rossin, A. Milella, L. Luconi, M. Innocenti, L.T. Phuoc, D.V. Cuong, P.H. Cuong, G. Giambastian, Chemically Functionalized Carbon Nanotubes with Pyridine Groups as Easily Tunable N-Decorated Nanomaterials for the Oxygen Reduction Reaction in Alkaline Medium, *Chem. Mat.*, **2014**, 26, 3460–3470.

© 2019 by the authors. Submitted for possible open access publication under the terms and conditions of the Creative Commons Attribution (CC BY) license (http://creativecommons.org/licenses/by/4.0/).
